# Utility of the C-Reactive Protein–Albumin–Lymphocyte (CALLY) Index in Prognostic Assessment of Pancreatic Head Adenocarcinomas Undergoing Pancreaticoduodenectomy

**DOI:** 10.3390/medicina62081517

**Published:** 2026-08-06

**Authors:** Kubilay Furkan Işıker, Ahmet Gökhan Sarıtaş, Uğur Topal, İshak Aydın, Serdar Gümüş, Burak Yavuz, Abdullah Ülkü, Atılgan Tolga Akçam

**Affiliations:** 1 Department of General Surgery, Faculty of Medicine, Cukurova University, Adana 01330, Türkiye; drags0001@hotmail.com (A.G.S.); sutopal2005@hotmail.com (U.T.); seredargumus@hotmail.com (S.G.); byvz@hotmail.com (B.Y.); gca_ulku@hotmail.com (A.Ü.); tlgakcam@yahoo.com (A.T.A.); 2 Department of Surgical Oncology, Faculty of Medicine, Cukurova University, Adana 01330, Türkiye; drishakaydin@gmail.com

**Keywords:** CALLY index, pancreatic adenocarcinoma, pancreaticoduodenectomy, prognosis

## Abstract

*Background and Objectives:* Systemic inflammation, nutritional status, and host immune response play pivotal roles in cancer progression and patient prognosis. The C-reactive protein–albumin–lymphocyte (CALLY) index is a novel inflammation-based biomarker integrating these parameters. This study aimed to evaluate the prognostic value of the preoperative CALLY index in patients undergoing pancreaticoduodenectomy for pancreatic head adenocarcinoma. *Materials and Methods:* This retrospective study included 101 consecutive patients who underwent pancreaticoduodenectomy with curative intent for histopathologically confirmed pancreatic head adenocarcinoma at Çukurova University Faculty of Medicine between 2010 and 2021. Demographic, clinicopathological, perioperative, and survival data were analyzed. The CALLY index was calculated using preoperative serum C-reactive protein, albumin, and lymphocyte levels, and the optimal cutoff value was determined by receiver operating characteristic (ROC) curve analysis. *Results:* Using an optimal cutoff value of 1.38, patients were categorized into low (*n* = 58) and high (*n* = 43) CALLY index groups. Patients with a low CALLY index experienced significantly higher rates of intraoperative and postoperative complications, surgical site infection, reoperation, unplanned hospital readmissions, and 30- and 90-day mortality (all *p* < 0.05). Moreover, the low CALLY index group demonstrated significantly poorer overall survival than the high CALLY index group (*p* < 0.001). Receiver operating characteristic (ROC) analysis for overall mortality yielded an area under the curve (AUC) of 0.701 (95% CI, 0.591–0.811). On multivariable Cox regression, a low CALLY index remained an independent predictor of poorer overall survival (adjusted hazard ratio [aHR], 2.45; 95% CI, 1.47–4.08; *p* < 0.001) after adjustment for age, tumor size, lymph node status, resection margin, and differentiation. On multivariable logistic regression, a low CALLY index was independently associated with surgical site infection (adjusted odds ratio [aOR], 11.5; 95% CI, 3.9–34.2; *p* < 0.001) and overall postoperative morbidity (aOR, 5.3; 95% CI, 2.0–14.0; *p* < 0.001). *Conclusions:* The preoperative C-reactive protein–albumin–lymphocyte (CALLY) index is a simple, inexpensive, and readily available biomarker with significant prognostic value in patients undergoing pancreaticoduodenectomy for pancreatic head adenocarcinoma. A low CALLY index was associated with poorer overall survival and increased postoperative morbidity and mortality. The CALLY index may serve as a practical tool for preoperative risk stratification and prognostic assessment. However, prospective multicenter studies are warranted to validate standardized cutoff values and further establish its clinical applicability.

## 1. Introduction

Pancreatic cancer remains one of the most aggressive malignancies worldwide and continues to be associated with an extremely poor prognosis despite advances in diagnostic and therapeutic strategies. Among its histological subtypes, pancreatic head adenocarcinoma accounts for the majority of resectable cases and is characterized by rapid disease progression, high metastatic potential, and limited long-term survival [1]. Because early-stage disease is frequently asymptomatic or accompanied by nonspecific clinical manifestations, many patients are diagnosed at an advanced stage, restricting curative treatment options. For patients with resectable pancreatic head adenocarcinoma, pancreaticoduodenectomy (Whipple procedure) remains the standard treatment with curative intent. Nevertheless, postoperative outcomes are highly heterogeneous and are influenced by multiple clinicopathological factors, including patient characteristics, tumor stage, lymph node involvement, and resection margin status [2,3].

Persistent inflammation is now recognized as a fundamental hallmark of cancer biology, contributing not only to tumor initiation but also to tumor growth, invasion, metastatic dissemination, and resistance to anticancer therapies. The interaction between malignant cells and the host inflammatory response creates a complex tumor microenvironment that promotes angiogenesis, immune evasion, and disease progression. Consequently, both systemic and local inflammatory responses have gained considerable attention as important determinants of prognosis across a wide range of solid malignancies [4].

Given the close relationship between systemic inflammation, nutritional status, and antitumor immunity, increasing attention has been directed toward composite inflammation-based biomarkers for prognostic assessment in oncology. Among these, the C-reactive protein–albumin–lymphocyte (CALLY) index has emerged as a promising biomarker that integrates serum C-reactive protein (CRP), albumin, and peripheral lymphocyte count into a single score reflecting the patient’s inflammatory, nutritional, and immunological status. Unlike biomarkers based on a single laboratory parameter, the CALLY index provides a more comprehensive assessment of host–tumor interactions and has demonstrated promising prognostic value in predicting postoperative outcomes, treatment response, and overall survival across a broad spectrum of malignancies. Owing to its simplicity, cost-effectiveness, and widespread availability in routine clinical practice, the CALLY index represents a practical tool for preoperative risk stratification and individualized patient management [5].

Since it was first described by Iida et al. [6] in patients undergoing hepatectomy for hepatocellular carcinoma, the CALLY index has been evaluated across a broad spectrum of malignancies—including gastric, colorectal, oral cavity, and biliary tract cancers—and, more recently, in non-oncological inflammatory settings, where a lower index has consistently been associated with greater systemic inflammation, poorer nutritional status, and adverse outcomes. It is therefore important to emphasize that the CALLY index is not specific to pancreatic cancer; rather, it reflects a generalizable host inflammatory, nutritional, and immune state, and its prognostic performance and optimal threshold must be validated within each tumor type and clinical context [5,6,7,8,9,10,11].

Although the CALLY index has shown promising prognostic value in several malignancies, its clinical significance in patients undergoing pancreaticoduodenectomy for pancreatic head adenocarcinoma remains insufficiently investigated. Therefore, this study aimed to evaluate the prognostic value of the preoperative CALLY index in patients undergoing pancreaticoduodenectomy for pancreatic head adenocarcinoma. Furthermore, we investigated its association with postoperative morbidity, short-term mortality, and overall survival to determine its potential role as a simple, readily available, and clinically applicable biomarker for perioperative risk stratification and postoperative management [5].

## 2. Materials and Method

This single-center retrospective study was approved by the Çukurova University Research Ethics Committee (Decision No. 31, dated 6 September 2024). All procedures were conducted in accordance with the ethical principles of the Declaration of Helsinki and relevant institutional guidelines and regulations. The study included consecutive adult patients who underwent pancreaticoduodenectomy with curative intent for histopathologically confirmed pancreatic head adenocarcinoma at the Department of General Surgery, Çukurova University Faculty of Medicine, between January 2010 and January 2021. Clinical data were retrospectively collected from the hospital electronic medical record system, archived patient files, prospectively maintained institutional databases, nursing records, operative reports, and pathology reports. Patients were eligible if they were ≥18 years of age, had undergone pancreaticoduodenectomy for resectable pancreatic head adenocarcinoma, and had complete preoperative laboratory, clinicopathological, operative, and follow-up data available for analysis. Patients were excluded if they had received neoadjuvant therapy, underwent palliative surgical procedures, had non-adenocarcinoma pancreatic head tumors, chronic inflammatory, autoimmune, hematological, or active infectious diseases that could influence inflammatory biomarkers, were pregnant, or had incomplete clinical data. A total of 101 patients fulfilled the eligibility criteria and were included in the final analysis. Eligible patients were consecutive adults (≥18 years) with resectable, histopathologically confirmed pancreatic head adenocarcinoma who underwent pancreaticoduodenectomy with curative intent and had complete preoperative laboratory, clinicopathological, operative, and follow-up data. To minimize the influence of non-tumoral inflammation on the CALLY index, patients with clinical or laboratory evidence of acute cholangitis, active biliary or systemic infection, or acute pancreatitis at the time of preoperative blood sampling were excluded, in addition to those with chronic inflammatory, autoimmune, hematological, or active infectious disease, prior neoadjuvant therapy, palliative or non-adenocarcinoma resections, and pregnancy. Preoperative endoscopic retrograde cholangiopancreatography (ERCP) with biliary stenting and percutaneous biliary drainage were systematically recorded, and their potential influence on inflammatory markers and the calculated CALLY index was assessed analytically (see Section Statistical Analysis).

The CALLY index was calculated as the product of the serum albumin concentration (g/L) and peripheral blood lymphocyte count (×10^9^/L), divided by the serum C-reactive protein (CRP) concentration (mg/L) multiplied by 10.

Preoperative blood samples were collected within seven days prior to surgery, either during hospital admission or at outpatient evaluation, and were used to calculate the CALLY index. Patients were subsequently stratified into two groups according to the optimal cutoff value determined by receiver operating characteristic (ROC) curve analysis: Group 1 (low CALLY index) and Group 2 (high CALLY index). Comparative analyses were performed to evaluate differences in demographic characteristics, body mass index (BMI), American Society of Anesthesiologists (ASA) score, preoperative laboratory findings, preoperative biliary interventions (including endoscopic retrograde cholangiopancreatography [ERCP]-guided biliary stenting and percutaneous biliary drainage), intraoperative variables, postoperative complications, blood transfusion requirements, reoperation rates, unplanned readmissions, length of hospital stay, pathological findings, and overall survival between the two groups.

Unplanned readmission was defined as any unplanned hospital admission occurring within 30 days after discharge from the index hospitalization. Reoperation was defined as any additional surgical procedure performed under general, spinal, or epidural anesthesia within 30 days after the index operation, excluding planned procedures based on pathological findings. Surgical site infections (SSIs) were classified according to the Centers for Disease Control and Prevention (CDC) criteria. Postoperative pancreatic fistula (POPF), postpancreatectomy hemorrhage (PPH), and delayed gastric emptying (DGE) were defined according to the criteria of the International Study Group of Pancreatic Surgery (ISGPS), where applicable [12]. Intraoperative blood transfusion was administered to patients with a hemoglobin (Hb) level <8 g/dL or to those who experienced a decrease in Hb of ≥3 g/dL from the preoperative baseline due to intraoperative blood loss. Postoperative blood transfusion was administered to patients with an Hb level <9 g/dL or to those with clinically significant postoperative bleeding.

All pancreaticoduodenectomies were performed by the same experienced surgical team using either the classic Whipple procedure or the pylorus-preserving pancreaticoduodenectomy technique. A reverse L-shaped incision was used in all patients, followed by reconstruction with duct-to-mucosa pancreaticojejunostomy, hepaticojejunostomy, and gastrojejunostomy. Tumor staging was determined according to the 8th edition of the American Joint Committee on Cancer (AJCC) TNM staging system. To ensure consistency throughout the study period, all pathological reports were retrospectively reviewed, and tumor staging was reassessed according to the AJCC 8th edition criteria. Reoperations were limited to surgical procedures performed within 30 days of the index operation to manage complications directly related to pancreaticoduodenectomy.

### Statistical Analysis

Statistical analyses were conducted using the SPSS 30.0 software package. The predictive value of the CALLY index for survival was assessed through receiver operating characteristic (ROC) curve analysis, which also determined the optimal cutoff value. Based on this cutoff, patients were categorized into two groups: those with a low CALLY index and those with a high CALLY index. Categorical data were presented as percentages (numbers), while continuous variables were reported as mean ± standard deviation for normally distributed parameters or median (interquartile range) for non-normally distributed parameters. Intergroup differences were evaluated using the chi-square test for categorical variables, the independent samples *t*-test for continuous variables with normal distributions, and the Mann–Whitney U test for non-normally distributed continuous variables. Survival outcomes were analyzed using the Kaplan–Meier method, and survival curves were compared between the groups. A *p*-value of <0.05 was considered statistically significant for all tests. The endpoint for ROC curve analysis was overall postoperative mortality; the area under the curve (AUC) is reported with its 95% confidence interval (CI), and sensitivity, specificity, predictive values, likelihood ratios (positive likelihood ratio [LR+] = sensitivity/[1 − specificity]; negative likelihood ratio [LR−] = [1 − sensitivity]/specificity), and overall accuracy were calculated at the optimal cutoff. To determine whether the CALLY index provided prognostic information independent of established clinicopathological factors, overall survival was analyzed using univariable and multivariable Cox proportional hazards regression, with results expressed as hazard ratios (HRs) and 95% CIs; the proportional hazards assumption was verified using Schoenfeld residuals. Variables associated with survival on univariable analysis and established prognostic factors (age, tumor size, lymph node status, resection margin, and differentiation) were entered into the multivariable model. Independent predictors of postoperative morbidity were identified using multivariable binary logistic regression, with results expressed as odds ratios (ORs) and 95% CIs. For outcomes with no events in the high CALLY group (30- and 90-day mortality), associations were assessed using Fisher’s exact test, because complete separation precluded stable regression estimates. Finally, the potential confounding effect of preoperative biliary drainage was explored by comparing CRP, albumin, lymphocyte count, bilirubin, and the CALLY index between patients with and without preoperative ERCP-guided stenting using the Mann–Whitney U test.

## 3. Results

**Figure 1:**
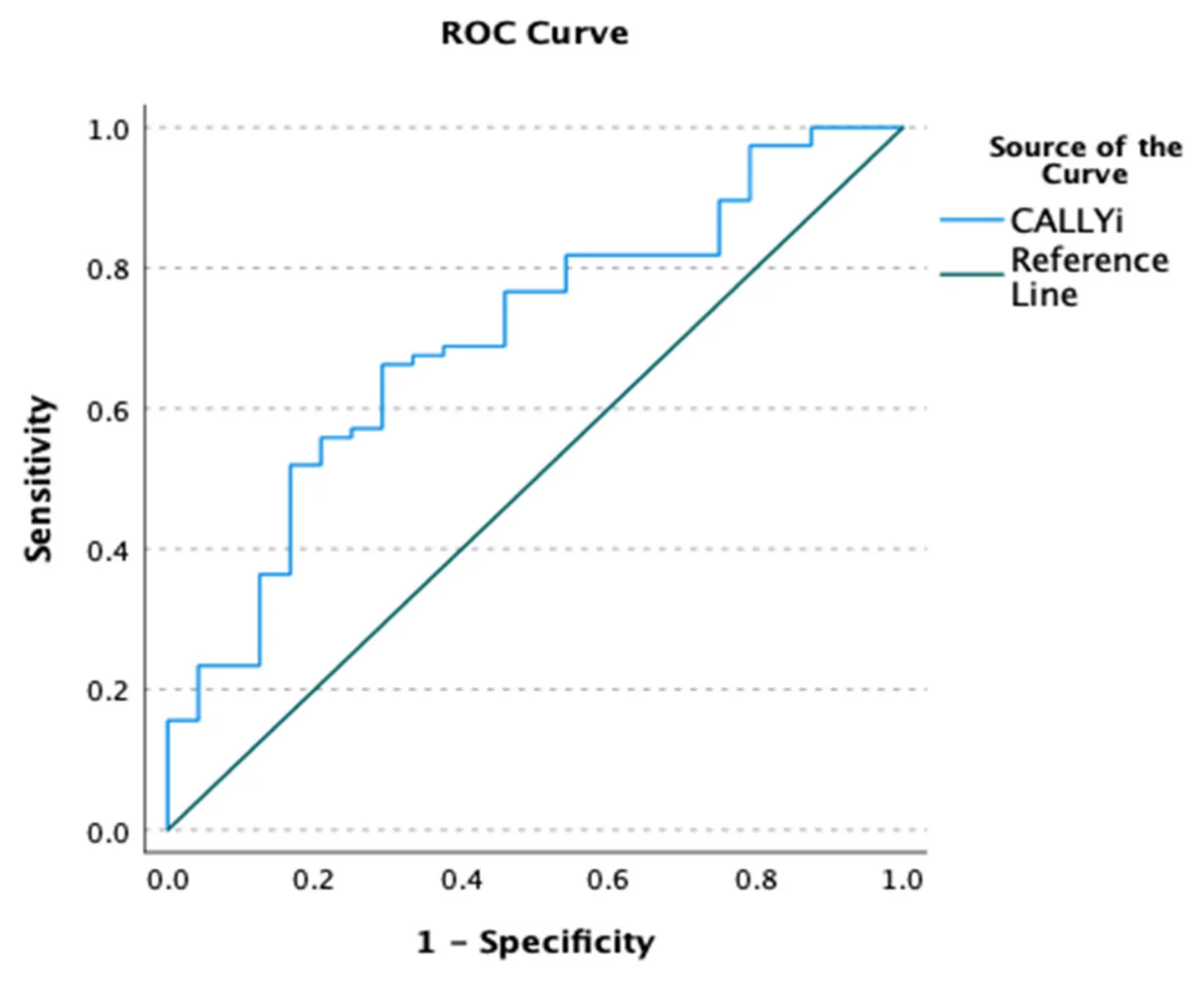
ROC Curve Analysis of the CALLY Index for Survival. Endpoint: overall postoperative mortality (AUC 0.701; 95% CI, 0.591–0.811; *p* = 0.001).

To establish a cutoff value for the CALLY index, a receiver operating characteristic (ROC) curve analysis was performed based on survival outcomes. The area under the ROC curve (AUC) was calculated at 70.1%, indicating that the derived cutoff value accurately predicted survival outcomes with 70.1% accuracy. The optimal CALLY index cutoff was determined to be 1.38. A CALLY index of <1.38 predicted survival with a sensitivity of 66.2% and a specificity of 70.8% (Table 1, Figure 1). The AUC was 0.701 (95% CI, 0.591–0.811; *p* = 0.001). At this cutoff, the positive and negative predictive values were 88% and 40%, the positive and negative likelihood ratios were 2.27 and 0.48, respectively, and the overall accuracy was 67.3%.

A total of 101 patients were included in the study and stratified into two groups according to the predefined CALLY index cutoff value: the low CALLY group (*n* = 58) and the high CALLY group (*n* = 43). Patients in the low CALLY group were significantly older than those in the high CALLY group (65.2 ± 8.7 vs. 60.8 ± 13.2 years, *p* = 0.040). No significant differences were observed between the groups regarding sex distribution, ASA score, BMI, serum CEA, CA19-9 levels, or other baseline clinical characteristics (Table 2). For the overall cohort, the median age was 65 years (range, 21–88); 52 patients (51.5%) were ≥65 years and 29 (28.7%) were ≥70 years of age.

Patients in the low CALLY group experienced significantly worse postoperative outcomes than those in the high CALLY group. Intraoperative complications were more frequent in the low CALLY group (19.0% vs. 2.3%, ***p*** = 0.011). Although postoperative pancreatic fistula occurred only in the low CALLY group (6.9% vs. 0%), this difference did not reach statistical significance (***p*** = 0.080). In contrast, biliary fistula (13.8% vs. 0%, ***p*** = 0.011), surgical site infection (62.1% vs. 14.0%, ***p*** < 0.001), and reoperation (15.5% vs. 2.3%, ***p*** = 0.028) were all significantly more common in patients with a low CALLY index. Likewise, both 30-day (17.2% vs. 0%, ***p*** = 0.004) and 90-day mortality rates (20.7% vs. 0%, ***p*** = 0.001) were significantly higher in the low CALLY group. Furthermore, the 3-year overall survival rate was markedly lower in patients with a low CALLY index than in those with a high CALLY index (17.2% vs. 60.5%, ***p*** < 0.001) (Table 3).

Because preoperative biliary obstruction and its drainage may influence systemic inflammatory markers, we examined whether preoperative ERCP-guided biliary stenting affected the components of the CALLY index. As expected, patients who underwent preoperative stenting had significantly higher serum bilirubin levels than those who did not (median, 2.59 vs. 0.83 mg/dL; *p* = 0.001). However, preoperative stenting was not associated with significant differences in CRP (median, 6.89 vs. 7.35 mg/L; *p* = 0.67), lymphocyte count (*p* = 0.57), albumin (*p* = 0.09), or the calculated CALLY index (median, 1.07 vs. 0.71; *p* = 0.76). Moreover, the proportion of patients undergoing preoperative stenting was comparable between the low and high CALLY groups (56.9% vs. 58.1%; *p* = 0.901). These findings suggest that, in this cohort, preoperative biliary drainage did not substantially confound the CALLY index.

On multivariable binary logistic regression adjusted for age, sex, and ASA score, a low CALLY index was independently associated with a markedly increased risk of surgical site infection (aOR, 11.5; 95% CI, 3.9–34.2; *p* < 0.001), unplanned readmission (aOR, 6.8; 95% CI, 1.8–26.2; *p* = 0.005), and overall postoperative complications (aOR, 5.3; 95% CI, 2.0–14.0; *p* < 0.001) (Table 4). Because 30- and 90-day mortality occurred exclusively in the low CALLY group, which precluded stable multivariable modelling, these associations were confirmed using Fisher’s exact test (90-day mortality: 20.7% vs. 0%; *p* = 0.001).

No significant differences were observed between the low and high CALLY groups regarding pathological tumor stage distribution according to the AJCC 8th edition TNM classification (*p* > 0.05). Likewise, surgical resection margin status (R0/R1) and tumor differentiation grade were comparable between the two groups, with no statistically significant differences (Table 5).

**Figure 2:**
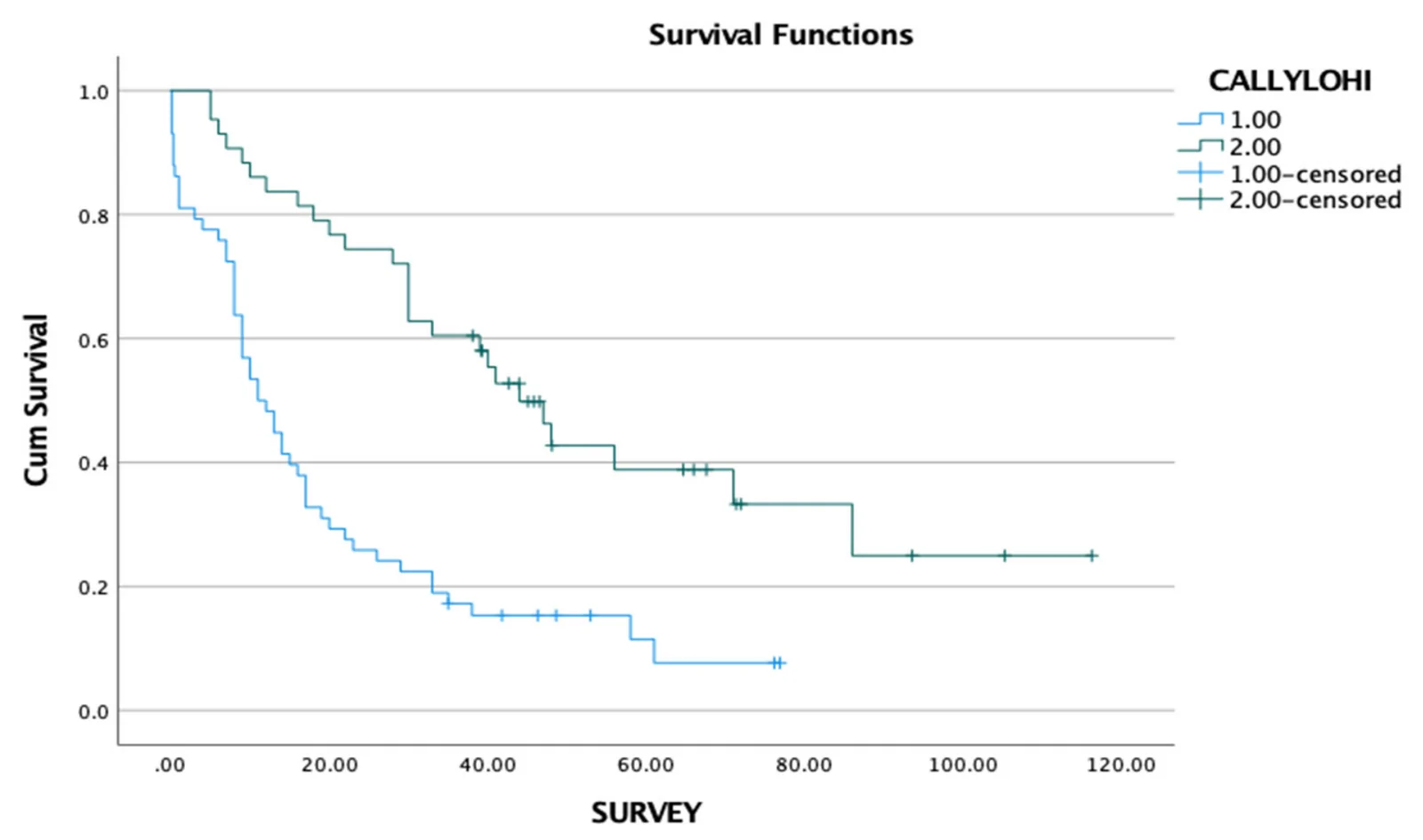
Kaplan–Meier Analysis of Overall Survival According to CALLY Index Groups.

Overall survival was significantly shorter in the low CALLY group than in the high CALLY group. The mean overall survival was 20.45 ± 3.01 months in the low CALLY group and 57.27 ± 6.88 months in the high CALLY group, while the overall mean survival for the entire cohort was 37.88 ± 4.23 months. This difference was statistically significant (*p* < 0.001) (Table 6, Figure 2).

To determine whether the CALLY index provided prognostic information independent of established clinicopathological factors, univariable and multivariable Cox proportional hazards analyses were performed (Table 7). On univariable analysis, a low CALLY index (HR, 2.89; 95% CI, 1.77–4.71; *p* < 0.001), older age, larger tumor size, poor differentiation, and R1/R2 resection were each associated with poorer overall survival. On multivariable analysis, a low CALLY index remained an independent predictor of poorer overall survival (aHR, 2.45; 95% CI, 1.47–4.08; *p* < 0.001), together with older age (aHR, 1.03 per year; 95% CI, 1.00–1.05; *p* = 0.026) and larger tumor size (aHR, 1.31 per cm; 95% CI, 1.10–1.57; *p* = 0.003).

**Table 7:** Univariable and multivariable Cox proportional hazards analysis for overall survival.

Variable	Univariable HR (95% CI)	*p*	Multivariable aHR (95% CI)	*p*
Low CALLY index (vs. high)	2.89 (1.77–4.71)	<0.001	2.45 (1.47–4.08)	<0.001
Age (per year)	1.03 (1.01–1.05)	0.008	1.03 (1.00–1.05)	0.026
Tumor size (per cm)	1.41 (1.19–1.68)	<0.001	1.31 (1.10–1.57)	0.003
Lymph node metastasis	1.21 (0.77–1.90)	0.411	1.08 (0.67–1.73)	0.756
R1/R2 resection	3.28 (1.16–9.22)	0.025	1.30 (0.43–3.94)	0.639
Poor differentiation	2.20 (1.15–4.22)	0.018	1.79 (0.88–3.64)	0.110

## 4. Discussion

The present study demonstrated that a low preoperative CALLY index was associated with significantly poorer postoperative and oncological outcomes in patients undergoing pancreaticoduodenectomy for pancreatic head adenocarcinoma. Specifically, patients with a low CALLY index experienced higher rates of intraoperative complications, biliary fistula, surgical site infections, reoperations, and both 30-day and 90-day mortality. Moreover, the low CALLY group exhibited significantly worse long-term survival than the high CALLY group. Although patients in the low CALLY group were significantly older, no significant differences were observed between the groups regarding other baseline demographic or clinicopathological characteristics, including ASA score, BMI, CEA, and CA19-9 levels. These findings suggest that the preoperative CALLY index may serve as a valuable prognostic biomarker for risk stratification in patients with pancreatic head adenocarcinoma undergoing pancreaticoduodenectomy.

The relationship between systemic inflammatory response, nutritional status, and cancer prognosis has been well-documented in the literature [13,14,15,16]. C-reactive protein (CRP) serves as a key indicator of inflammatory activity within the tumor microenvironment. Concurrently, albumin, a marker of nutritional status, is often diminished in cancer patients due to heightened metabolic demands. Serum albumin provides a straightforward measure of visceral protein reserves, with hypoalbuminemia reflecting both malnutrition and systemic inflammation. Several studies have identified serum albumin as an independent predictor of survival and prognosis in cancer patients [17,18]. Tumor-associated inflammation fosters stromal remodeling, immunosuppression, and metastatic progression. Lymphocytes, essential components of innate and adaptive immunity, play a central role in immune surveillance and antitumor activity [19]. Tumor-infiltrating lymphocytes, particularly CD8+ and CD4+ T-cells, are integral to initiating antitumor immune responses, including the induction of apoptosis in malignant cells [20]. Conversely, low lymphocyte counts are indicative of impaired antitumor immunity, and preoperative lymphocyte levels have been positively correlated with survival outcomes in numerous studies [21,22].

Recognizing the interplay between systemic inflammation, nutritional status, and host immunity, the C-reactive protein–albumin–lymphocyte (CALLY) index was developed as a novel composite biomarker. Owing to its simplicity, cost-effectiveness, and widespread availability, the CALLY index has attracted considerable attention as a prognostic indicator in oncology [5]. Zhang et al. demonstrated that the CALLY index was a superior prognostic biomarker in patients with gastric cancer, further supporting its potential clinical applicability across different malignancies [7]. Similarly, Tsai et al. first demonstrated its clinical utility in patients with oral cavity cancer, reporting that a low preoperative CALLY index was independently associated with poorer overall survival [8].

Iida et al. (2022) [6] evaluated the prognostic significance of the CALLY index in patients undergoing hepatectomy for hepatocellular carcinoma. Patients with a CALLY index <5 had a significantly higher proportion of advanced-stage disease (TNM stage III/IV) than those with a CALLY index ≥5 (*p* = 0.003). Moreover, the 5-year overall survival rate was significantly lower in the low CALLY group than in the high CALLY group (46% vs. 71%, *p* < 0.001), further supporting the prognostic value of the CALLY index in solid malignancies [6].

Tsunematsu et al. [9] examined the prognostic impact of the CALLY index in patients with distal cholangiocarcinoma undergoing pancreaticoduodenectomy and identified an optimal cutoff value of 3.5. Among the 87 patients with a preoperative CALLY index <3.5 (61%), multivariable analysis demonstrated that obstructive jaundice requiring drainage (***p*** < 0.01), poorly differentiated tumors (***p*** < 0.01), and a CALLY index <3.5 (***p*** = 0.02) were independent predictors of both disease-free survival (DFS) and overall survival (OS) [9]. Similarly, Zhang et al. (2023) [7] evaluated the prognostic significance of the CALLY index in gastric cancer and established a cutoff value of 1.12. Patients with a CALLY index >1.12 exhibited significantly better overall survival than those with a CALLY index ≤1.12 (***p*** < 0.0001) [7]. Consistent with these findings, our study demonstrated that patients in the high CALLY index group had significantly improved overall survival following pancreaticoduodenectomy for pancreatic head adenocarcinoma (***p*** < 0.01).

Yang et al. [10] investigated the prognostic significance of the CALLY index in patients with colorectal cancer and demonstrated that a lower CALLY index was independently associated with poorer overall survival. In addition to survival, patients with lower CALLY values exhibited more advanced tumor characteristics and an unfavorable inflammatory profile, suggesting that the CALLY index reflects not only tumor biology but also the host inflammatory and nutritional status. These findings are consistent with our results, in which patients with a low CALLY index experienced significantly poorer postoperative outcomes and overall survival following pancreaticoduodenectomy [10]. In contrast, our study did not identify a significant association between the CALLY index and TNM stage or differentiation grade (*p* > 0.05) in pancreatic head adenocarcinoma. However, a higher proportion of Stage IIA+ tumors (62.1%) was observed in the low CALLY index group, suggesting that a potential predictive relationship may become evident with larger cohorts. Importantly, the low CALLY index group demonstrated significantly lower 3-year survival rates (*p* < 0.001), reinforcing the prognostic relevance of the CALLY index in pancreatic head adenocarcinoma.

Fukushima et al. (2024) [11] evaluated the prognostic significance of the CALLY index in 826 patients undergoing gastrectomy for gastric cancer and identified an optimal cutoff value of 2. Patients with a preoperative CALLY index <2 had significantly poorer overall survival and recurrence-free survival. Furthermore, a low CALLY index remained an independent predictor of survival outcomes after adjustment for other clinicopathological factors, highlighting its prognostic value beyond conventional risk factors [11]. Consistent with these findings, the CALLY index was also a significant prognostic marker in our cohort. Patients with a low CALLY index experienced significantly higher rates of surgical site infection, intraoperative and postoperative complications, reoperation, early postoperative mortality, and unplanned hospital readmissions, all of which contributed to inferior overall survival. Collectively, these findings support the utility of the CALLY index for identifying high-risk patients and optimizing perioperative management.

In addition to the CALLY index, other biomarkers, such as the HALP (hemoglobin, albumin, lymphocyte, platelet) score, neutrophil-to-lymphocyte ratio (NLR), and platelet-to-lymphocyte ratio (PLR), have been investigated for their prognostic significance in pancreatic cancer. Studies suggest that these indices are significant predictors of clinical outcomes. For example, Xu et al. [23] demonstrated that low HALP scores in resected pancreatic adenocarcinoma patients were associated with early recurrence (7.3 vs. 16.3 months RFS, *p* < 0.001) and shorter OS (11.5 vs. 23.6 months, *p* < 0.001). Furthermore, low HALP was identified as an independent risk factor for poor outcomes, irrespective of gender or tumor location [23]. Similarly, our findings align with these observations, as a low CALLY index was significantly correlated with reduced survival (*p* < 0.01).

On the other hand, despite the growing evidence supporting the prognostic value of the CALLY index, several limitations remain before its widespread clinical implementation. A recent systematic review and meta-analysis highlighted that the currently available evidence is largely based on retrospective studies, predominantly involving East Asian populations, with considerable variability in the optimal cutoff values used across different cancer types. These limitations restrict the generalizability of current findings and underscore the need for large prospective multicenter studies to validate standardized cutoff values and further establish the clinical utility of the CALLY index [24].

Collectively, the existing literature and our findings consistently demonstrate that the CALLY index is closely associated with perioperative outcomes and long-term prognosis across various gastrointestinal malignancies. In our cohort, a low preoperative CALLY index was associated with increased postoperative morbidity and mortality, as well as significantly poorer overall survival following pancreaticoduodenectomy. These findings further support the clinical utility of the CALLY index as a simple, inexpensive, and readily available biomarker for preoperative risk stratification and prognostic assessment in patients with pancreatic head adenocarcinoma.

Importantly, the prognostic value of the CALLY index in the present cohort was independent of established clinicopathological factors. On multivariable Cox regression, a low CALLY index remained significantly associated with poorer overall survival after adjustment for age, tumor size, lymph node status, resection margin, and differentiation (aHR, 2.45; 95% CI, 1.47–4.08), and on multivariable logistic regression, it was an independent predictor of surgical site infection and overall postoperative morbidity. These findings indicate that the CALLY index captures prognostic information beyond conventional staging and provide the multivariable evidence that earlier single-marker analyses lacked.

Patients in the low CALLY group were significantly older than those in the high CALLY group. Aging is accompanied by immunosenescence—characterized by a decline in naïve T-cell output and circulating lymphocyte counts together with a chronic, low-grade proinflammatory state (“inflammaging”)—which may lower the CALLY index independently of tumor burden and could partly explain the observed age difference between groups. Reassuringly, the CALLY index retained independent prognostic significance after adjustment for age, indicating that its effect is not merely a surrogate for chronological age; nevertheless, age-related immune remodeling should be considered when interpreting the index in elderly surgical candidates.

The evidence supporting the CALLY index, although broadly favorable, is not uniform. Reported optimal cutoff values vary substantially across tumor types and populations—for example, 1.12 in gastric cancer, 2 in gastrectomy cohorts, 3.5 in distal cholangiocarcinoma, and 5 in hepatocellular carcinoma—reflecting differences in assay methods, patient case-mix, and the absence of a standardized formula. Some analyses have found the index to be attenuated or non-significant after multivariable adjustment or to be outperformed by other composite markers such as the HALP score, and a recent systematic review and meta-analysis cautioned that the current evidence is dominated by retrospective, single-center studies from East Asian populations. Our results should therefore be interpreted within this heterogeneous and still-maturing body of literature, and the cutoff of 1.38 derived here should be regarded as cohort-specific pending external validation.

A further consideration specific to pancreatic head tumors is the frequent presence of obstructive jaundice and preoperative biliary drainage, which could theoretically elevate CRP and thereby lower the CALLY index independently of tumor biology. In our cohort, however, preoperative ERCP-guided stenting was not associated with significant differences in CRP or the calculated CALLY index, and its distribution was balanced across CALLY groups, suggesting that biliary intervention did not materially confound the index in this setting. This observation nonetheless warrants confirmation in larger cohorts with standardized timing of blood sampling relative to biliary drainage.

This study has several limitations. First, its retrospective, single-center design may have introduced selection bias and limited the generalizability of our findings. Second, although patients with active infection were excluded whenever possible, preoperative laboratory parameters used to calculate the CALLY index may have been influenced by unrecognized inflammatory conditions or biliary obstruction, potentially affecting the accuracy of the index. Third, the CALLY index was calculated using only preoperative laboratory data, and changes in inflammatory and nutritional status during the postoperative period were not evaluated. Furthermore, as highlighted by a recent systematic review and meta-analysis, most studies investigating the CALLY index are retrospective, were conducted predominantly in East Asian populations, and employed different cutoff values across various malignancies, limiting the generalizability and clinical applicability of the current evidence. Therefore, large prospective multicenter studies are warranted to validate standardized cutoff values and further validate the prognostic value and clinical applicability of the CALLY index. Several additional limitations warrant emphasis. The modest single-center sample size (*n* = 101) limits statistical power and raises the possibility of both type I error (spurious associations arising from multiple comparisons) and type II error (failure to detect true associations, as suggested by the non-significant trend for pancreatic fistula, which occurred only in the low CALLY group). The cohort was drawn from a single tertiary referral center serving a defined geographic region of Türkiye, and the resulting ethnic and referral homogeneity may limit generalizability to other populations. The prolonged inclusion period, during which chemotherapy regimens, perioperative care pathways, and surgical technique evolved, may have introduced temporal heterogeneity in outcomes. The optimal cutoff was both derived and tested within the same cohort without external validation, and survival times for censored patients were reconstructed from operative and last-contact records, which may introduce minor imprecision in the survival estimates. Finally, the exclusive occurrence of early postoperative mortality in the low CALLY group precluded multivariable adjustment for the 30- and 90-day mortality endpoints, which were therefore assessed only by exact testing.

## 5. Conclusions

The present study demonstrated that the preoperative CALLY index is a valuable prognostic biomarker in patients undergoing pancreaticoduodenectomy for pancreatic head adenocarcinoma. A low CALLY index was associated with poorer overall survival, increased postoperative morbidity, and higher postoperative mortality, highlighting its potential role in preoperative risk stratification and prognostic assessment. Given its simplicity, low cost, and widespread availability, the CALLY index may serve as a practical adjunct to routine preoperative evaluation. Nevertheless, prospective multicenter studies involving larger and more diverse patient populations are needed to validate standardized cutoff values and further establish the prognostic value and clinical applicability of the CALLY index.

## Figures and Tables

**Table 1:** Cut-off Value of the CALLY Index for Survival. Endpoint for ROC analysis was overall postoperative mortality. AUC is reported with its 95% confidence interval; likelihood ratios were calculated from sensitivity and specificity.

Parameter	Value
ROC (AUC)	0.701 (95% CI, 0.591–0.811)
*p*-value	**0.001**
Cut-off Value	1.38
Sensitivity	66.2%
Specificity	70.8%
Positive Predictive Value (PPV)	88%
Negative Predictive Value (NPV)	40%
Positive Likelihood Ratio (LR+)	2.27
Negative Likelihood Ratio (LR−)	0.48
Accuracy	67.3%

Bold font indicates statistically significant results (*p* < 0.05).

**Table 2:** Demographic and Preoperative Findings Based on CALLY Index Groups. Data are presented as mean ± SD, median (interquartile range), or *n* (%). Groups were compared using Student’s *t*-test, Mann–Whitney U test, or chi-square/Fisher’s exact test, as appropriate.

Parameter	Low CALLY (*n* = 58)	High CALLY (*n* = 43)	*p*-Value
**Age (Mean ± SD) (Min-Max)**	65.2 ± 8.7	60.8 ± 13.2	**0.04**
**Sex**			0.404
Male	56.9% (33)	65.1% (28)	
Female	43.1% (25)	34.9% (15)	
**ASA Score**			0.249
1	3.4% (2)	7.0% (3)	
2	79.3% (46)	86.0% (37)	
3	17.2% (10)	7.0% (3)	
**BMI (Median, IQR)**	25 (23.3–27.3)	26.8 (24.1–28.3)	0.083
**CEA (Median, IQR)**	2.59 (1.72–4.88)	2.48 (1.55–3.41)	0.313
**CA19.9 (Median, IQR)**	150.6 (46.3–415.0)	103.7 (11.7–305.1)	0.133
**Preop WBC (Mean ± SD)**	7900.0 ± 990.0	7860.0 ± 937.0	0.530
**Preop Plt (Mean ± SD)**	289,500 ± 35,000	305,000 ± 38,900	0.471
**Preop T. Bil (Mean ± SD)**	1.96 ± 0.80	1.00 ± 0.57	0.211
**Preop Amylase (Mean ± SD)**	66.0 ± 39.0	74.0 ± 51.0	0.154
**Preop ALP (Mean ± SD)**	164.0 ± 103.0	135.0 ± 94.0	0.548
**Preop GGT (Mean ± SD)**	147.0 ± 44.0	94.0 ± 31.0	0.193
**Preop CRP (Mean ± SD)**	15.55 ± 10.30	1.56 ± 0.67	**<0.001**
**Preop Albumin (Mean ± SD)**	31 ± 2.6	36 ± 3.2	**<0.001**
**Preop Lymphocyte (Mean ± SD)**	1.80 ± 1.20	2.24 ± 1.68	**0.009**
**Preop Hgb (Mean ± SD)**	12.2 ± 1.6	12.4 ± 1.5	0.293
**Preop Hct (Mean ± SD)**	35.90 ± 4.43	36.72 ± 4.29	0.176

Bold *p* values indicate statistical significance (*p* < 0.05).

**Table 3:** Preoperative, Intraoperative, and Postoperative Characteristics Based on CALLY Index Groups. Data are presented as *n* (%) and compared using the chi-square or Fisher’s exact test.

Parameter	Low CALLY (*n* = 58)	High CALLY (*n* = 43)	*p*-Value
**Preop ERCP Stent**			0.901
-----Present	56.9% (33)	58.1% (25)	
-----Absent	43.1% (25)	41.9% (18)	
**Preop Percutaneous Drainage**			0.437
-----Present	8.6% (5)	4.7% (2)	
-----Absent	91.4% (53)	95.3% (41)	
**Intraoperative Complications**			**0.011**
-----Absent	81% (47)	97.7% (42)	
-----Present	19.0% (11)	2.3% (1)	
**Intraoperative Transfusion**			0.08
-----Absent	50% (29)	67.4% (29)	
-----Present	50% (29)	32.6% (14)	
**Postoperative Transfusion**			0.110
-----Present	96.6% (56)	88.4% (38)	
-----Absent	3.4% (2)	11.6% (5)	
**Postoperative Complications**			**0.035**
-----Abscess	6.9% (4)	4.7% (2)	
-----Atony	24.1% (14)	9.3% (4)	
-----Evisceration	1.7% (1)	0	
-----Ileus	1.7% (1)	0	
-----Gastroenterostomy Leak	3.4% (2)	0	
-----Hemorrhage	12.1% (7)	2.3% (1)	
-----Absent	50% (29)	83.7% (36)	
**Pancreatic Fistula**	6.9% (4)	0	0.08
**Biliary Fistula**	13.8% (8)	0	**0.011**
**Surgical Site Infection**	62.1% (36)	14.0% (6)	**<0.001**
**Reoperation Required**	15.5% (9)	2.3% (1)	**0.028**
**Unplanned Readmission**	31.0% (18)	7.0% (3)	**0.003**
**30-Day Mortality**	17.2% (10)	0	**0.004**
**90-Day Mortality**	20.7% (12)	0	**0.001**
**3-Year Survival**	17.2% (10)	60.5% (26)	**<0.001**

Bold *p* values indicate statistical significance (*p* < 0.05).

**Table 4:** Multivariable binary logistic regression analysis for postoperative morbidity (adjusted for age, sex, and ASA score).

Postoperative Outcome	Adjusted OR for Low CALLY İndex (95% CI)	*p*-Value
Surgical site infection	11.5 (3.9–34.2)	<0.001
Unplanned readmission	6.8 (1.8–26.2)	0.005
Any postoperative complication	5.3 (2.0–14.0)	<0.001

**Table 5:** Comparison of Pathological Findings According to the CALLY Index. Staging follows the AJCC 8th edition; data are *n* (%), compared using the chi-square/Fisher’s exact test.

Parameter	Low CALLY (*n* = 58)	High CALLY (*n* = 43)	*p*-Value
**Tumor Localization**			-
Pancreatic Head	58 (100.0%)	43 (100.0%)	-
**Stage**			0.687
IA	7 (12.1%)	8 (18.6%)	
IB	15 (25.9%)	8 (18.6%)	
IIA	4 (6.9%)	3 (7.0%)	
IIB	20 (34.5%)	18 (41.9%)	
III	12 (20.7%)	6 (14.0%)	
**Surgical Resection (R0)**			0.468
Present	55 (94.8%)	42 (97.7%)	
Absent	3 (5.2%)	1 (2.3%)	
**Differentiation**			0.800
Poor	9 (15.5%)	3 (7.0%)	
Well	25 (43.1%)	28 (65.1%)	
Moderate	24 (41.4%)	12 (27.9%)	

**Table 6:** Overall Survival According to the CALLY Index. Values are Kaplan–Meier mean survival estimates (months) with 95% CI; groups compared using the log-rank test.

CALLY Index	Mean Survival (95% CI)	*p*-Value
**Low**	20.450 (95% CI 14.553–26.347)	**<0.001**
**High**	57.273 (95% CI 43.789–70.757)	
**Overall**	37.879 (95% CI 29.592–46.166)	

Bold *p* values indicate statistical significance (*p* < 0.05).

## Data Availability

The original contributions presented in this study are included in the article. Further inquiries can be directed to the corresponding author.
